# Tubal ligation and risk of breast cancer

**DOI:** 10.1054/bjoc.1999.1182

**Published:** 2000-04-03

**Authors:** L A Brinton, M D Gammon, R J Coates, R N Hoover

**Affiliations:** Environmental Epidemiology Branch, Epidemiology and Biostatics Program, National Cancer Institute, Executive Plaza South, Rm. 7068, 6120 Executive Boulevard, MSC 7234, Bethesda, MD 20892-7234, USA; Department of Epidemiology, University of North Carolina, Chapel Hill, NC 27599-7400, USA; Centers for Disease Control and Prevention, Atlanta, GA 30341, USA

**Keywords:** breast cancer, tubal ligation, epidemiology

## Abstract

Although it has been demonstrated in previous studies that tubal ligation can have widespread effects on ovarian function, including a decrease in the risk of subsequent ovarian cancer, few studies have evaluated effects on breast cancer risk. In a population-based case–control study of breast cancer among women 20–54 years of age conducted in three geographic areas, previous tubal ligations were reported by 25.3% of the 2173 cases and 25.8% of the 1990 controls. Initially it appeared that tubal ligations might impart a slight reduction in risk, particularly among women undergoing the procedure at young ages (< 25 years). However, women were more likely to have had the procedure if they were black, less educated, young when they bore their first child, or multiparous. After accounting for these factors, tubal ligations were unrelated to breast cancer risk (relative risk (RR) = 1.09, 95% confidence interval (CI) 0.9–1.3), with no variation in risk by age at, interval since, or calendar year of the procedure. The relationship of tubal ligations to risk did not vary according to the presence of a number of other risk factors, including menopausal status or screening history. Furthermore, effects of tubal ligation were similar for all stages at breast cancer diagnosis. Further studies would be worthwhile given the biologic plausibility of an association. However, future investigations should include information on type of procedure performed (since this may relate to biologic effects) as well as other breast cancer risk factors. © 2000 Cancer Research Campaign


*British Journal of Cancer* (2000) **82**(9), 1600–1604
© 2000 Cancer Research Campaign
DOI: 10.1054/ bjoc.1999.1182, available online at http://www.idealibrary.com on IDEAL®

### Tubal ligation and risk of breast cancer

**LA Brinton[1], MD Gammon[3], RJ Coates[4] and RN Hoover[2]**

[1]Environmental Epidemiology Branch and [2]Epidemiology and Biostatics Program, National Cancer Institute, Executive Plaza South, Rm. 7068, 6120 Executive Boulevard, MSC 7234, Bethesda, MD 20892-7234, USA; [3]Department of Epidemiology, University of North Carolina, Chapel Hill, NC 27599-7400, USA; [4]Centers for Disease Control and Prevention, Atlanta, GA 30341, USA

**Summary** Although it has been demonstrated in previous studies that tubal ligation can have widespread effects on ovarian function, including a decrease in the risk of subsequent ovarian cancer, few studies have evaluated effects on breast cancer risk. In a population-based case–control study of breast cancer among women 20–54 years of age conducted in three geographic areas, previous tubal ligations were reported by 25.3% of the 2173 cases and 25.8% of the 1990 controls. Initially it appeared that tubal ligations might impart a slight reduction in risk, particularly among women undergoing the procedure at young ages (< 25 years). However, women were more likely to have had the procedure if they were black, less educated, young when they bore their first child, or multiparous. After accounting for these factors, tubal ligations were unrelated to breast cancer risk (relative risk (RR) = 1.09, 95% confidence interval (CI) 0.9–1.3), with no variation in risk by age at, interval since, or calendar year of the procedure. The relationship of tubal ligations to risk did not vary according to the presence of a number of other risk factors, including menopausal status or screening history. Furthermore, effects of tubal ligation were similar for all stages at breast cancer diagnosis. Further studies would be worthwhile given the biologic plausibility of an association. However, future investigations should include information on type of procedure performed (since this may relate to biologic effects) as well as other breast cancer risk factors. © 2000 Cancer Research Campaign

**Keywords**: breast cancer; tubal ligation; epidemiology

Studies have recently demonstrated that tubal ligation results in a significant reduction in the subsequent risk of ovarian cancer (Hankinson et al, 1993; Rosenblatt et al, 1996; Green et al, 1997*a*; Kreiger et al, 1997; Miracle-McMahill et al, 1997). Selective screening for ovarian abnormalities during the procedure cannot account for the striking deficits in risk (40–60%) that have been observed. Alternative explanations include possible effects of reducing ovarian blood supply, destroying tissue at risk, or reducing exposure of the ovaries to exogenous or endogenous factors that may be involved in ovarian cancer development.

It has recently been hypothesized that breast cancer risk may also be reduced by tubal ligation. It is well known that ovarian ablation substantially reduces breast cancer risk, presumably because of striking decreases in endogenous hormones (La Vecchia, 1999). Two recent reports have shown reductions in breast cancer following tubal ligations (Calle et al, 1999; Kreiger et al, 1999). Surprisingly, few other epidemiologic investigations have assessed the relationship of tubal ligations to breast cancer risk, with one supporting the hypothesis of reduced risk (Shin et al, 1996) and the other showing no such protective effect (Irwin et al, 1988).

A large investigation of breast cancer among younger women, many of whom reported such procedures, enabled an evaluation of tubal procedures in relation to risk independent of other risk factors.

*Received 6 October 1999*
*Revised 26 October 1999*
*Accepted 24 October 1999*

*Correspondence to:* LA Brinton

### MATERIALS AND METHODS

This population-based case–control study was conducted in three different geographic areas – the metropolitan areas of Atlanta, Georgia and Seattle/Puget Sound, Washington, and five counties of central New Jersey. In Seattle and New Jersey, the study was confined to women 20–44 years of age, while in Atlanta the age range was extended through age 54. All women of these ages newly diagnosed with in situ or invasive breast cancer during the period 1 May 1990 to 31 December 1992 were identified through rapid ascertainment systems. All areas were covered by population-based cancer registries, and periodic checks against these registries determined the completeness of case ascertainment. Hospital records of eligible patients were examined for details on the clinical and pathologic characteristics of the diagnosed breast cancers.

Controls in the three geographic areas were ascertained through a series of 13 waves of random digit dialing (Waksberg, 1978). To select a sample of women whose ages approximated to the anticipated age distribution of cases, information was sought on female residents who were 20–44 years of age (20–54 years in Atlanta). A 90.5% response rate to the telephone screener was obtained from the 16 254 telephone numbers assessed as residential; non-response consisted of a 5.4% refusal to the telephone screener, 0.8% language problems and 3.3% contact problems.

Structured in-person interviews (median 67 min) covered demographic factors; reproductive and menstrual history; contraceptive behaviour; use of exogenous hormones; medical and screening history; anthropometry and physical activity; adolescent diet; alcohol consumption; smoking; occupations; family history of cancer; and certain lifestyle factors and opinions about cancer causation. Subjects were also asked to complete a 100-item dietary questionnaire and to consent to a variety of anthropometric measurements.

Subjects were asked to complete a month-by-month calendar documenting all contraceptive methods used since menarche. Pregnancies and other life events were first marked on the calendar to serve as a frame of reference for changes in contraceptive behaviour over time. Information recorded on the calendar regarding the occurrence of a tubal ligation was used to compute the age at, interval since, and calendar time of the procedure.

Completed interviews were obtained from 2202 of the 2558 eligible cases (86.1%) and 2009 of the 2477 eligible controls (81.1%). Reasons for non-interview included subject refusals (6.4% in cases vs 14.0% in controls), death (0.4% vs 0.2%), illness (0.6% vs 0.2%), language problems (0.3% vs 1.4%), a move outside of the study area (0.6% vs 2.3%) and other miscellaneous reasons (0.2% vs 0.8%). In addition, physician consent for interview was denied for 5.4% of the cases. Among controls, an overall response rate of 73.4% was achieved through multiplication of the telephone screener and interview response rates. To assure comparability between the cases and controls, the 29 cases who indicated on interview that they did not have a residential telephone and the 19 controls with a history of breast cancer were eliminated, leaving 2173 cases and 1990 controls available for analysis.

Since the median interval between diagnosis and interview was 87 days for cases, all information on risk factors, including that pertaining to tubal ligations, was truncated at the date of diagnosis for cases or the date at completion of the telephone screener for controls. The relationship of breast cancer risk factors to tubal ligation among the controls was assessed by calculating $\chi^2$ statistics. The relationship of tubal ligation to breast cancer risk was assessed through calculation of odds ratios to approximate relative risks (RRs). Logistic regression analyses were used to obtain maximum likelihood estimates of RRs and their 95% confidence intervals (CI) (Breslow and Day, 1980). The significance of interactions of variables was determined by using multiplicative terms in the regression models, as described by Thompson (1994).

**Table 1** Per cent of controls reporting a previous tubal ligation by selected risk factors

| Risk factor | Number of controls | % Reporting previous tubal | $\chi^2$ *P*-value |
|---|---|---|---|
| Site | | | |
| Atlanta | 919 | 28.1 | |
| New Jersey | 462 | 21.2 | |
| Seattle | 609 | 25.9 | 0.023 |
| Race | | | |
| White | 1555 | 23.5 | |
| Black | 323 | 37.5 | |
| Other | 112 | 25.0 | 0.001 |
| Education | | | |
| High school or less | 586 | 34.1 | |
| Post high school | 162 | 29.0 | |
| Some college | 509 | 27.7 | |
| College graduate | 467 | 16.5 | |
| Post graduate | 266 | 18.4 | 0.001 |
| Income | | | |
| < $15 000 | 161 | 28.6 | |
| $15–24 999 | 209 | 24.4 | |
| $25–34 999 | 284 | 27.8 | |
| $35–49 999 | 388 | 26.0 | |
| $50–69 999 | 372 | 26.1 | |
| $70–89 999 | 239 | 29.3 | |
| $90 000+ | 284 | 20.4 | |
| Unknown | 53 | 22.6 | 0.372 |
| Number of births | | | |
| 0 | 392 | 3.8 | |
| 1 | 362 | 14.1 | |
| 2 | 645 | 30.2 | |
| 3 | 369 | 41.5 | |
| 4+ | 222 | 45.0 | 0.001 |
| Age at first birth | | | |
| <20 | 375 | 37.6 | |
| 20–24 | 574 | 38.3 | |
| 25–29 | 406 | 26.6 | |
| 30+ | 242 | 12.0 | |
| Nulliparous | 392 | 3.8 | 0.001 |
| Previous mammogram | | | |
| No | 869 | 23.5 | |
| Yes | 1120 | 27.7 | 0.088 |

### RESULTS

A total of 25.3% of the cases versus 25.8% of the controls reported a prior tubal ligation. Among the control subjects, tubal ligation rates were highest in Atlanta (28.1%) and lowest in New Jersey (21.2%). Previous analyses in this study population have shown elevations in breast cancer risk associated with White race, a first-degree family history of breast cancer, a previous breast biopsy, nulliparity, a late age at first birth, lower body mass, extended use of oral contraceptives and heavy consumption of alcoholic beverages (Brinton et al, 1995; Swanson et al, 1996, 1997). Control subjects were more likely to report a previous tubal ligation if they were black, less educated, young when they bore their first child, or multiparous. In addition, tubal ligations were more common among subjects who had been screened by mammography, particularly those with multiple mammograms (data not shown). In contrast, the prevalence of tubal ligations did not appear to be related to type of menopause, income, body mass index, years of use of exogenous hormones, or alcohol consumption (data not shown).

Table 2 presents relative risks associated with various aspects of a previous tubal ligation. The risk for ever having had a tubal ligation, adjusted only for the frequency matching factors of study site and age, was 0.95. Further adjustment for race, age at first birth, number of births and years of education increased this risk to 1.09 (95% CI 0.9–1.3), with the main confounder being late age at first birth. Adjustment for additional risk factors (including mammographic screening history) did not further affect the risk. The majority of subjects had their operations after the age of 30. Although there was no variation in risk by age at operation for procedure performed after the age of 25, the unadjusted analysis suggested that operations prior to this age reduced breast cancer risk. However, these women also had young ages at first birth and, after adjustment for this as well as other factors, the reduction in risk was attenuated (RR = 0.94, 95% CI 0.6-1.4). Neither interval since nor calendar year of operation was predictive of risk. Risk was not altered even among subjects with recent (< 2 years) or distant (≥ 15 years) operations.

Given the slight reduction in risk experienced by women who had their operations at young ages, we assessed risk in relation to some combined timing variables, including a cross-classification of age at and interval since tubal ligation; little variation was found. The RRs associated with the procedure prior to age 30 were 1.00 (95% CI 0.6–1.6) and 1.20 (0.9–1.6) for those with < 10 and 10+ years since the surgery respectively; with ligation at 30 years of age or older, comparable risks were 1.06 (0.9–1.3) and 1.07 (0.8–1.4).

© 2000 Cancer Research Campaign

**Table 2** Relative risks of breast cancer by particulars of a previous tubal ligation

| | Cases | Controls | Unadjusted RR[a] | Adjusted RR[b] | 95% CI |
|---|---|---|---|---|---|
| Ever had a tubal ligation | | | | | |
| No | 1624 | 1476 | 1.00 | 1.00 | |
| Yes | 549 | 514 | 0.95 | 1.09 | 0.9–1.3 |
| Age at tubal ligation | | | | | |
| < 25 | 39 | 51 | 0.72 | 0.94 | 0.6–1.4 |
| 25–29 | 141 | 127 | 1.02 | 1.23 | 0.9–1.6 |
| 30–34 | 184 | 179 | 0.92 | 1.04 | 0.8–1.3 |
| 35+ | 185 | 157 | 1.03 | 1.09 | 0.9–1.4 |
| Years since tubal ligation | | | | | |
| < 5 | 102 | 89 | 1.08 | 1.23 | 0.9–1.7 |
| 5–9 | 144 | 148 | 0.88 | 0.97 | 0.8–1.2 |
| 10–14 | 182 | 177 | 0.91 | 1.07 | 0.8–1.4 |
| 15+ | 121 | 100 | 1.03 | 1.29 | 0.9–1.7 |
| Calendar year of tubal ligation | | | | | |
| < 1975 | 86 | 64 | 1.13 | 1.40 | 0.9–2.0 |
| 1975–1979 | 149 | 158 | 0.83 | 0.97 | 0.8–1.2 |
| 1980–1984 | 155 | 172 | 0.81 | 0.91 | 0.7–1.2 |
| 1985+ | 159 | 120 | 1.23 | 1.35 | 1.0–1.8 |

[a]Adjusted for study site and age. [b]Adjusted for study site, age, combination of age at first birth and number of births, and years of education.

**Table 3** Relative risks of breast cancer associated with a previous tubal ligation by levels of other risk factors

| | Cases | | Controls | | | |
|---|---|---|---|---|---|---|
| | Unexposed | Exposed | Unexposed | Exposed | RR[a] | 95% CI |
| Race | | | | | | |
| Whites | 1326 | 390 | 1190 | 365 | 1.08 | 0.9–1.3 |
| Non-Whites | 298 | 159 | 286 | 149 | 1.14 | 0.8–1.6 |
| Site | | | | | | |
| Atlanta | 747 | 284 | 661 | 258 | 1.13 | 0.9–1.4 |
| New Jersey | 394 | 115 | 364 | 98 | 1.03 | 0.7–1.4 |
| Seattle | 483 | 150 | 451 | 158 | 0.98 | 0.7–1.3 |
| Age | | | | | | |
| < 35 | 239 | 29 | 255 | 36 | 0.61 | 0.3–1.1 |
| 35–39 | 386 | 101 | 370 | 104 | 1.06 | 0.7–1.5 |
| 40–44 | 622 | 270 | 500 | 236 | 1.06 | 0.8–1.3 |
| 45+ | 377 | 149 | 351 | 138 | 1.17 | 0.9–1.6 |
| Age at first birth | | | | | | |
| < 20 | 183 | 132 | 234 | 141 | 1.27 | 0.9–1.8 |
| 20–24 | 351 | 211 | 354 | 220 | 0.95 | 0.7–1.2 |
| 25–29 | 343 | 119 | 298 | 108 | 0.91 | 0.6–1.2 |
| 30+ | 281 | 56 | 213 | 29 | 1.38 | 0.8–2.3 |
| Nulliparous | 465 | 31 | 377 | 15 | 1.49 | 0.8–2.8 |
| Menopausal status | | | | | | |
| Premenopausal | 1307 | 431 | 1156 | 389 | 1.02 | 0.8–1.2 |
| Menopausal, intact ovaries | 120 | 38 | 126 | 51 | 1.15 | 0.7–2.0 |
| Menopausal, ovaries removed | 186 | 74 | 177 | 68 | 1.24 | 0.8–1.9 |
| Previous mammogram | | | | | | |
| No | 628 | 181 | 665 | 204 | 0.98 | 0.8–1.3 |
| Yes | 996 | 368 | 810 | 310 | 1.13 | 0.9–1.4 |

[a]Relative risks pertain to the risk associated with tubal ligation within strata of selected risk factors. Risks are adjusted for study site, age, years of education, and, where appropriate, for number of births and age at first birth.

The relationship of tubal ligations showed little variation according to other breast cancer risk factors (Table 3). A lower risk was observed for younger subjects (< 35 years of age at breast cancer diagnosis) (RR = 0.61), while a slightly higher risk was observed for nulliparous women (RR = 1.49). Both of these risks were based on relatively small numbers and neither was statistically different than the null. Risks associated with a tubal ligation were similar in subjects who reported never versus ever having had a previous mammogram. Similar risks associated with a tubal ligation were seen across different menopause categories. Of note was that there was no differential relationship of tubal ligations among women who subsequently had a bilateral oophorectomy, despite this operation leading to a significant reduction in breast cancer risk in this population (RR = 0.59, 95% CI 0.4–0.8).

*© 2000 Cancer Research Campaign*

The stage distribution of tumours was 15.4% in situ, 47.2% stage 1, 35.7% stage 2 or greater, and 1.8% missing. The RRs for tubal ligation did not vary significantly by stage, being 1.34 (95% CI 1.0–1.8) for in situ tumours, 1.10 (95% CI 0.9–1.3) for stage 1 cancers and 1.03 (95% CI 0.8–1.3) for stage 2+ disease. Given that the risk for in situ tumours was elevated, we further assessed this according to timing but no distinctive pattern was found; in particular, we did not observe the highest risk for operations performed recently, which would have supported the notion of a detection bias.

### DISCUSSION

Our finding that tubal ligation is not associated with a reduced risk of breast cancer is at variance with several other investigations. In a large record linkage study involving 268 423 women with tubal ligations, the procedure was associated with a statistically significant incidence ratio of 0.84 (Kreiger et al, 1999). Tubal ligations also appeared to reduce risk (RR = 0.37) in a small case–control study in Korea (Shin et al, 1996). However, in both studies no relationship was found with age at or interval since tubal ligation, arguing against causality. Recently, a 12-year mortality follow-up study, based on 3086 breast cancer deaths, observed a rate ratio of 0.76 (Calle et al, 1999). Risks were lowest among those sterilized before age 35 and prior to 1975, suggesting that tissue damage with early procedures may have been involved.

Similar to this latest study, we initially observed that women undergoing tubal ligations at young ages (< 25 years) were at reduced risk, but this relationship did not persist after adjustment for effects of age at first birth. Similar confounding of tubal ligation effects by reproductive behaviour has been noted in an endometrial cancer study (Castellsague et al, 1996). One of the few previous breast cancer studies which was able to adjust for other risk factors observed tubal ligation to be associated with a slight increase in risk (RR = 1.2), although no relation was found with age at or time since surgery (Irwin et al, 1988). This was in contrast to their findings that bilateral oophorectomy decreased risk, possibly by curtailing ovarian function at a critical time. Methodologic differences may be relevant to determine why our results diverge from the large investigations. Although one of these studies (Kreiger et al, 1999) was not able to control for confounding, it is noteworthy that even our unadjusted risks did not reflect any reduction in risk. In the study by Calle and others (Calle et al, 1999), breast cancer mortality was the end point rather than incidence, as in our study. Thus, if patients with tubal ligations are more intensively screened, as we observed, it is possible that a spuriously protective effect with mortality would be apparent. However, we did not observe a reduction in risk even among subjects with advanced stage disease. Another difference included our focus on younger women (< 55 years of age). Although the studies that found a reduced risk associated with tubal ligation generally focused on women over 50 years of age, we saw no reduced risk associated with tubal ligation even among our oldest subjects. In contrast, a reduced risk was found among the youngest women (< 35 years) in our study.

Unfortunately, neither our study nor any of the previous investigations had information on the type of procedures performed. These range from techniques performed by laparotomy (mainly the Pomeroy technique) to those performed by laparoscopy (unipolar coagulation, bipolar coagulation, Yoon Fallope rings, Hulka-Clemens clips), which can have different biologic effects, particularly on blood flow and tissue damage (Donnez et al, 1981). Differences between investigations may have reflected variations in the procedures employed, which we could assess only indirectly and crudely.

Strengths of our study included a large sample size, high rate of exposure to tubal ligation, and the ability to consider effects of other breast cancer risk factors. However, given the case-control design of our study, it is possible that some subjects may have misreported their histories of tubal ligation. Any mis-reporting would have affected our results primarily if cases and controls were not equally likely to report their histories (differential misclassification) (Armstrong, 1998). It is more likely that our results would have been influenced by non-differential misclassification, which can bias results towards the null. Although we were unable to evaluate the extent to which mis-classification affected results, it has been found elsewhere that tubal ligations are accurately reported (Green et al, 1997b).

Although we found no significant effect of tubal ligation on breast cancer risk, the issue appears to deserve further investigation, especially given the biologic credibility of a link with breast cancer risk. This includes clinical reports showing that menstrual disorders (Neil et al, 1975; Sorenson and Ladehoff, 1979; DeStefano et al, 1985) and alterations in oestrogen and progesterone levels (Cattanach and Milne, 1988; Helm amd Sjoberg, 1983; Hakverdi et al, 1994) have been seen following tubal ligations. Future studies involving prospective designs may be most useful, given the potential in case–control studies, such as ours, for difficulties in recall. These studies will need to consider the influence of other breast cancer risk factors (notably reproductive behaviour) and to obtain information on the types of tubal ligations performed.

### REFERENCES

Armstrong BG (1998) Effect of measurement error on epidemiological studies of environmental and occupational exposures. *Occup Environ Med* **55**: 641–646

Breslow NE and Day NE (1980) *Statistical Methods in Cancer Research. Vol. 1. The Analysis of Case-control Studies*. IARC Sci. Pub. No. 32. International Agency for Research on Cancer: Lyon

Brinton LA, Daling JR, Liff J, Schoenberg JB, Malone KE, Stanford JL, Coates RJ, Gammon MD, Hanson L and Hoover RN (1995) Oral contraceptives and breast cancer risk among younger women. *J Natl Cancer Inst* **87**: 827–835

Calle E, Rodriguez C, Walker K and Thun M (1999) Tubal sterilization and breast cancer mortality in a prospective cohort of US women. *Am J Epidemiol* **149**: S27 (abstract)

Castellsague X, Thompson WD and Dubrow R (1996) Tubal sterilization and the risk of endometrial cancer. *Int J Cancer* **65**: 607–612

Cattanach JF and Milne BJ (1988) Post-tubal sterilization problems correlated with ovarian steroidogenesis. *Contraception* **38**: 541–550

DeStefano F, Perlman JA, Peterson HB and Diamond EL (1985) Long-term risk of menstrual disturbances after tubal sterilization. *Am J Obstet Gynecol* **152**: 835–841

Donnez J, Wauters M and Thomas K (1981) Luteal function after tubal sterilization. *Obstet Gynecol* **57**: 65–68

Green A, Purdie D, Bain C, Siskind V, Russell P, Quinn M, Ward B and Survey of Women's Health Study Group (1997*a*) Tubal sterilisation, hysterectomy and decreased risk of ovarian cancer. *Int J Cancer* **71**: 948–951

Green A, Purdie D, Green L, Dick ML, Bain C and Siskind V (1997*b*) Validity of self-reported hysterectomy and tubal sterilisation. The Survey of Women's Health Study Group. *Aust NZ J Public Health* **21**: 337–340

Hakverdi AU, Taner CE, Erden AC and Satici Ö (1994) Changes in ovarian function after tubal sterilization. *Adv Contracept* **10**: 51–56

Hankinson SE, Hunter DJ, Colditz GA, Willett WC, Stampfer MJ, Rosner B, Hennekens CH and Speizer FE (1993) Tubal ligation, hysterectomy, and risk of ovarian cancer. A prospective study. *JAMA* **270**: 2813–2818

© 2000 Cancer Research Campaign

Helm G and Sjoberg N-O (1983) Progesterone levels before and after laparoscopic tubal sterilization using endotherm coagulation. *Acta Obstet Gynecol Scand* **62**: 63–66

Irwin KL, Lee NC, Peterson HB, Rubin GL, Wingo PA, Mandel MG and Cancer and Steroid Hormone Study Group (1988) Hysterectomy, tubal sterilization, and the risk of breast cancer. *Am J Epidemiol* **127**: 1192–1201

Kreiger N, Sloan M, Cotterchio M and Parson P (1997) Surgical procedures associated with risk of ovarian cancer. *Int J Epidemiol* **26**: 710–715

Kreiger N, Sloan M, Cotterchio M and Kirsh V (1999) The risk of breast cancer following reproductive surgery. *Eur J Cancer* **35**: 97–101

La Vecchia C (1999) Reproductive surgery, menopause and breast cancer risk. *Eur J Cancer* **35**: 12–13

Miracle-McMahill HL, Calle EE, Kosinski AS, Rodriguez C, Wingo PA, Thun MJ and Heath CW Jr (1997) Tubal ligation and fatal ovarian cancer in a large prospective cohort study. *Am J Epidemiol* **145**: 349–357

Neil JR, Noble AD, Hammond GT, Rushton L, Letchworth AT (1975) Late complications of sterilization by laparoscopy and tubal ligation. *Lancet* **2**: 699–700

Rosenblatt KA, Thomas DB, the World Health Organization Collaborative Study of Neoplasia and Steroid Contraceptives (1996) Reduced risk of ovarian cancer in women with a tubal ligation or hysterectomy. *Cancer Epidemiol Biomarkers Prev* **5**: 933–935

Shin MH, Kim DH, Lee HK, Yang JH, Choi KJ and Ahn YO (1996) The effect of tubal ligation on the protection from breast cancer: a case–control study in Korea (Abstract). *Am J Epidemiol* **143**: S58

Sorenson T and Ladehoff P (1979) Late complication of sterilization in women. *Ugeskr Laeger* **141**: 998–999

Swanson CA, Coates RJ, Schoenberg JB, Malone KE, Gammon MD, Stanford JL, Shorr I, Potischman NA and Brinton LA (1996) Body size and breast cancer risk among women under age 45 years. *Am J Epidemiol* **143**: 698–706

Swanson CA, Coates RJ, Malone KE, Gammon MD, Schoenberg JB, Brogan DR, McAdams M, Potischman N, Hoover RN and Brinton LA (1997) Alcohol consumption and breast cancer risk among women under age 45. *Epidemiology* **8**: 231–237

Thompson WD (1994) Statistical analysis of case–control studies. *Epidemiol Rev* **16**: 33–50

Waksberg J (1978) Sampling methods for random digit dialing. *J Am Stat Assoc* **73**: 40–46

 *© 2000 Cancer Research Campaign*
